# Assessment of the PD-1/PD-L1/PD-L2 Immune Checkpoints Pathway in Endometrial Cancer and Its Clinical Significance

**DOI:** 10.3390/cancers17213485

**Published:** 2025-10-29

**Authors:** Karolina Włodarczyk-Ciekańska, Agnieszka Kwiatkowska-Makuch, Anna Pawłowska-Łachut, Wiktoria Skiba, Dorota Suszczyk, Jan Kotarski, Paulina Pieniądz-Feculak, Anna Pańczyszyn, Anna Ignatowicz, Rafał Tarkowski, Iwona Wertel

**Affiliations:** 1 Independent Laboratory of Cancer Diagnostics and Immunology, Faculty of Medicine, Medical University of Lublin, Chodźki 1, 20-093 Lublin, Poland; anna.pawlowska@umlub.pl (A.P.-Ł.); wiktoria.skiba@umlub.pl (W.S.); dorota.suszczyk@umlub.pl (D.S.); jan.kotarski.gabinet@gmail.com (J.K.); paulina.pieniadz@umlub.pl (P.P.-F.); iwona.wertel@umlub.pl (I.W.); 2 I Chair and Department of Oncological Gynecology and Gynaecology, Medical University of Lublin, 20-059 Lublin, Poland; kwiatkowska.makuch@gmail.com (A.K.-M.); rafal.tarkowski@umlub.pl (R.T.); 3 Department of Virology and Immunology, Institute of Biology and Biotechnology, Maria Curie-Skłodowska University, Akademicka 19, 20-031 Lublin, Poland; 4 Medical Department, Institute of Medical Sciences, University of Opole, Oleska 48, 45-052 Opole, Poland; apanczyszyn@uni.opole.pl; 5 Students’ Scientific Association, Independent Laboratory of Cancer Diagnostics and Immunology, Medical University of Lublin, Chodźki 1, 20-093 Lublin, Poland; an.ignatowicz@onet.pl

**Keywords:** endometrial cancer, immune checkpoints, PD-1/PD-L1/PD-L2, immunosuppression

## Abstract

**Background**: Endometrial cancer is one of the most common female genital cancers and poses a significant clinical problem due to its increasing incidence and variable prognosis depending on the stage of the disease. The development of EC is largely dependent on interactions with the immune system, including immune checkpoints (ICPs) such as PD-1, PD-L1, and PD-L2. The aim of our study was to evaluate the PD-1/PD-L1/PD-L2 pathway in EC and its clinical significance. **Methods**: The analysis was performed by flow cytometry on myeloid and plasmacytoid dendritic cells and monocytes (MO) in peripheral blood (PB). The concentration of sPD-1, sPD-L1, and sPD-L2 in plasma was determined by ELISA. Additionally, *PD-L1* and *PD-L2* gene expression levels in tumor tissue (TT) were assessed using real-time polymerase chain reaction (qPCR). The obtained results were correlated with clinical data of EC patients. **Results**: Patients with EC had lower percentages of PD-L1-positive MO and pDCs, as well as PD-L2-positive MO and mDCs, compared with the control group. We observed accumulation of sPD-1 and lower levels of sPD-L1 and sPD-L2 in EC patients compared to the control group, with sPD-L2 correlating with *PD-L2* gene expression level in the TT. **Conclusions**: The study results indicate a difference in the distribution of mDCs, pDCs, and MO with PD-L1/PD-L2 expression in EC patients. Reduced percentages of MO and DCs expressing PD-L1 and PD-L2, altered concentrations of soluble forms of these IPCs, and correlations with gene expression in TT suggest that dysregulation of this pathway may influence disease progression. Furthermore, the relationships between immunological parameters and clinical features such as BMI and FIGO stages suggest the potential use of these factors as diagnostic and prognostic biomarkers and the possibility of incorporating them into future therapeutic strategies. However, further studies are necessary to validate this hypothesis.

## Simple Summary

Endometrial cancer (EC) develops in an environment strongly modulated by the immune system, and changes in dendritic cells (mDCs and pDCs), and monocytes (MO) expressing PD-L1 and PD-L2 may influence disease progression. Our study demonstrated that EC patients have lower percentages of PD-L1-positive MO and pDCs, as well as PD-L2-positive MO and mDCs, compared to the control group. We also found changes in plasma levels of soluble forms of PD-1, PD-L1, and PD-L2, which correlated with tumor *PD-L2* expression and clinical characteristics, such as BMI and disease FIGO stage.

## 1. Introduction

Endometrial cancer (EC) is the sixth most common disease and the thirteenth most common cause of cancer deaths among women worldwide. According to the latest data from the World Health Organization (WHO), in 2022, EC was diagnosed in over 420,000 women, of whom nearly 98,000 patients died [1,2,3,4]. The highest rates of EC are observed in North America, where the age-standardized rate (ASR) is 22.5 per 100,000 females, and in Europe, where the ASR is 15.5 per 100,000 females [1]. A worrying and alarming fact is the constantly increasing mortality rates among patients with endometrial cancer for over two decades [4,5]. They mainly concern older women (over the age of 60) with a very aggressive form of EC.

EC primarily affects women over the age of 60 who have already gone through menopause. However, there are cases of women still menstruating around the age of 45, but they are rare and constitute about 7% of all cases [1,6,7,8]. EC is associated with risk factors such as lifestyle diseases (including obesity) and gynecological diseases (e.g., polycystic ovary syndrome—PCOS), exposure to estrogens, reproductive factors, childlessness, genetic predisposition (e.g., Lynch syndrome), lifestyle without physical activities or tamoxifen therapy [3,9,10,11].

For many years, the role of the immune system in a lot of malignancies, including EC, was overlooked. However, the knowledge about the biology of this cancer was very limited and did not improve the rates of morbidity or mortality. In recent years, a key role in the carcinogenesis of patients with EC has been attributed to the tumor microenvironment (TME) [12,13]. In the case of EC cells, immune evasion and progression may be attributed to the induction or inhibition of T lymphocyte cell death, which plays a crucial role in mounting an antitumor response through immune checkpoints (ICPs) [14,15].

Dendritic cells (DCs) also play an important role in the TME, bridging innate and adaptive immunity by presenting antigens and modulating T cell activity [16,17]. In tumor tissue (TT), their numbers are typically reduced, and their functions are inhibited by tumor cytokines, such as interleukin-10 (IL-10), resulting in the emergence of immune tolerance and the proliferation of regulatory T cells (Tregs). Myeloid DCs (mDCs), stimulated by tumor necrosis factor alpha (TNF-α), activate T cells, inducing tumor cell apoptosis, contributing to the support of an antitumor response. Plasmacytoid DCs (pDCs), on the other hand, exhibit immunosuppressive activity and may support tumor progression. DCs in the TME may be an important element for use in immunotherapy, by stimulating mDCs or inhibiting pDCs, enhancing the antitumor response, and increasing T cell activity [16,17,18].

One example of a key player in the negative regulation of T cells is the programmed cell death protein 1 (PD-1) axis with its ligands, programmed death-ligand 1 (PD-L1) and programmed death-ligand 2 (PD-L2). In cancer cells, ligands bind to PD-1 and interact with it, which suppresses T cell effector functions, thereby causing a lack of antitumor response. PD-L1 is expressed by some cells of the immune system (including T cells, macrophages, dendritic cells) and by cancer cells [12,13,15,16,19,20,21,22]. The presence of this ligand on cancer cells enables the inhibition of the T lymphocyte response by interacting with PD-1, facilitating immune evasion. In contrast, in the literature, there is much less information about the second ligand, PD-L2, which could also be expressed on antigen-presenting cells and cancer cells. Although its expression is lower than PD-L1, by binding to PD-1, it can enhance the effect of immunosuppression and promote tumor progression [13,14,20,21,23,24].

This axis allows cancer cells to remain undetected by the patient’s immune system. That is why the introduction of immunotherapy based on PD-L1 inhibitors in EC was such an important breakthrough [25,26]. In 2021, the Food and Drug Administration (FDA) approved the possibility of treating EC patients with Dostarlimab, which works by inhibiting the binding of PD-1 to ligands, through the interaction of Dostarlimab with PD-L1 and PD-L2, allowing T lymphocytes to activate and attack cancer cells [27,28,29,30,31].

The aim of our study was to assess the expression of PD-1/PD-L1/PD-L2 axis in endometrial cancer in relation to the clinicopathological features. Additionally, the level of soluble forms of PD-1, PD-L1, and PD-L2 in the plasma of patients with EC and the analysis of *PD-L1* and *PD-L2* gene expression levels in tumor tissue (TT) were assessed in correlation with clinicopathological features of EC patients, and compared to healthy blood donors and the reference group.

## 2. Materials and Methods

### 2.1. Patients and the Control Group

Our study involved 125 patients, including 32 healthy blood donors as the control group, 15 patients with Leiomyoma as the reference group, and 78 endometrial cancer patients, diagnosed and operated between 2021 and 2023 in the I Chair and Department of Oncological Gynaecology and Gynaecology, Medical University Clinical Hospital No. 1 in Lublin, Poland. The diagnosis of endometrioid EC was confirmed histopathologically and classified into one of four stages according to the International Federation of Gynecologists and Obstetricians (FIGO) classification. The exclusion criteria for the study cohort included a history of previous malignancies, chemotherapy, or radiation therapy prior to surgery, as well as allergic, autoimmune, and infectious diseases. Written informed consent was obtained from all EC patients. The research received approval of the Bioethics Committee of the Medical University of Lublin (KE-0254/212/2020). The control group was obtained through the Regional Centre of Blood Donation and Blood Treatments in Lublin and included 32 healthy blood donors, as well as the reference group consisting of patients with uterine fibroids. Every patient provided written consent to participate in the study, and the experiments were conducted in accordance with the Declaration of Helsinki. The clinical characteristics of EC patients are presented in Table 1.

### 2.2. Material

All peripheral blood (PB) samples were collected in potassium EDTA S-Monovette tubes (Sarstedt, Nümbrecht, Germany) before the surgical procedure. Tumor tissue (TT) was collected aseptically during surgery. Blood samples were centrifuged for 10 min at 1000 rpm at 4 °C to obtain plasma, which was stored at −80 °C until further analysis. PB mononuclear cells (PBMCs) were separated by density gradient centrifugation at 2800 rpm for 20 min at room temperature with Lymphoprep (Stemcell Technologies, Vancouver, BC, Canada). Then, the collected interphase cells were washed twice and suspended in phosphate-buffered saline without Ca^2+^ and Mg^2+^ (PBS, Capricorn Scientific, Ebsdorfergrund, Germany). The PBMC were isolated within 2 h of collection and used for the flow cytometry analysis.

The tumor tissue was fragmented into smaller pieces, then distributed in RNAlater (ThermoFischer Scientific, Waltham, MA, USA) and stored at −80 °C until further analysis.

### 2.3. Flow Cytometry Analysis

Cell staining was performed according to the manufacturer’s instructions in the presence of FcR blocking reagent (Miltenyi-Biotec, Bergisch Gladbach, Germany). To perform the flow cytometry analysis, the PBMCs (1 × 10^6^ cells) were transferred into tubes and incubated with a combination of the following monoclonal antibodies (mAbs) against cell-surface markers: anti-BDCA-1 FITC (Miltenyi-Biotec, Bergisch Gladbach, Germany), anti-CD19 PerCP-Cy5.5 (BD Biosciences, San Jose, CA, USA), anti-BDCA-2 FITC (Miltenyi-Biotec, Bergisch Gladbah, Germany), anti-CD123 PE-Cy7 (BioLegend, San Diego, CA, USA), anti-CD45 FITC (BD Biosciences, San Jose, CA, USA), anti-CD14 PE-Cy7 (BD Biosciences, San Jose, CA, USA), anti-PD-L1 APC (BioLegend, San Diego, CA, USA), and anti-PD-L2 PE (BioLegend, San Diego, CA, USA). Furthermore, the appropriate amount of aqua fluorescent reactive dye LIVE/DEAD Fixable Aqua Dead Cell Stain Kit (Thermo Fisher Scientific, Waltham, MA, USA) was added to the antibody cocktail. After incubation in the dark for 30 min (at room temperature, RT) and washing in 1% bovine serum albumin-enriched PBS, samples were directly analyzed by FACS. The immunolabelled cells were collected (a total of 100,000 events) using an FACSCanto I flow cytometer (BD Biosciences, San Jose, CA, USA) and analyzed with FACSDiva software v6.1.3.

**Figure 1:**
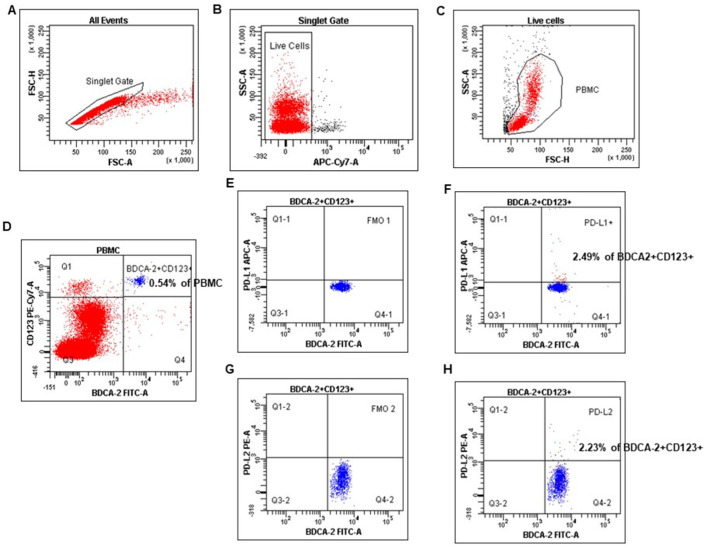
The identification of the plasmacytoid dendritic cell population in peripheral blood mononuclear cells (PBMC). PBMC were stained and acquired using FACS. pDCs were identified with the following gating strategy. (**A**) A combination of forward scatter area (FSC-A) and forward scatter height (FSC-H) was utilized to exclude doublets. (**B**) After gating for singlets, live cells were selected. (**C**) Next, mononuclear cells were selected based on their side-scatter/forward-scatter (SSC/FSC) properties. (**D**) Gate on the BDCA-2^+^CD123^+^ (positive) cell population in BDCA-2 FITC vs. CD123 PE-Cy7 dot plot. (**E**–**H**) Selected BDCA-2^+^CD123^+^ cells were analyzed for PD-L1 and PD-L2 expression. The final dot plot (**F**) indicates cells BDCA-2^+^CD123^+^PD-L1^+^ (PD-L1^+^ pDCs). The final dot plot (**H**) indicates cells BDCA-2^+^CD123^+^PD-L2^+^(PD-L2^+^ pDCs). The plot (**E**) is an FMO control for PD-L1 (FMO1), stained with all markers in the panel, except for PD-L1. The plot (**G**) is an FMO control for PD-L2 (FMO2), stained with all markers in the panel, except for PD-L2. Legend: red indicates PB mononuclear cells, and blue indicates %pDCs (BDCA-2^+^CD123^+^).

The numbers of myeloid and plasmacytoid DCs were quantified as the percentages of PB mononuclear cells, as described in our and other authors’ earlier published papers [32,33]. mDCs were defined as BDCA-1 positive and simultaneously CD19 negative cells, whereas pDCs as double BDCA-2 and CD123 positive cells (Figure 1). Cell debris and dead cells were excluded from the analysis based on scatter signals and LIVE/DEAD Fixable Aqua Dead Cell Stain Kit. The FcR Blocking Reagent has been used to avoid Fc receptor–mediated antibody labeling. Fluorescence minus one (FMO) control was used to verify the staining specificity and as a guide for setting the markers to delineate positive populations (mDCs, pDCs, and MO with PD-L1, PD-L2 expression).

### 2.4. ELISA

The concentration of the soluble forms of PD-1 (sPD-1), PD-L1 (sPD-L1) and PD-L2 (sPD-L2) were measured in the plasma of EC patients, as well as in the plasma of the control and reference group, using an ELISA immunoassay kits (sPD-1, Biorbyt LLC., San Francisco, CA, USA; detection range 15.6 to 1000 pg/mL; sensitivity 3.9 pg/mL sPD-L1, R&D Systems, Minneapolis, MN, USA; detection range 25 to 1600 pg/mL; sensitivity 4.52 pg/mL; sPD-L2, Invitrogen, Waltham, MA, USA; detection range 39 to 2500 pg/mL; sensitivity 3.65 pg/mL) following the manufacturers’ protocols. Plate absorbance was read on an ELX-800 plate reader (BioTek Instruments, Winooski, VT, USA) and analyzed by Gen5_ (BioTek Instruments, Winooski, VT, USA).

### 2.5. Nucleic Acid Extraction and cDNA Synthesis

Endometrial tissue biopsy samples were immersed in RNAlater reagent (Thermo Fischer Scientific, Waltham, MA, USA) and then stored in a freezer at −80 °C. Total RNA was isolated from frozen tissue using the TRIzol Reagent (Thermo Fischer Scientific, Waltham, MA, USA) according to the manufacturer’s instructions. Isolated RNA was purified using the Universal RNA Purification Kit (Eurx, Gdańsk, Poland). Then, RNA was reverse transcribed using the iScript cDNA Synthesis Kit (Bio-Rad Laboratories, Hercules, CA, USA) in a final volume of 20 µL according to the following protocol: 5 min at 25 °C, 20 min at 46 °C, and reverse transcriptase inactivation was performed for 1 min at 95 °C.

### 2.6. Quantitative PCR

The expression levels of *CD274 (PD-L1,* Assay ID: Hs00204257_m1) and *PDCD1LG2* (*PD-L2*, Assay ID: Hs01057777_m1) were measured using the TaqMan (Thermo Fisher Scientific, Waltham, MA, USA) real-time polymerase chain reaction (qPCR) method. The qPCR reaction was performed in a final volume of 10 µL containing 0.5 µL of 20× TaqMan Gene Expression Assay (Thermo Fischer Scientific, Waltham, MA, USA), 5 µL of 2× TaqMan Gene Expression Master Mix (Thermo Fischer Scientific, Waltham, MA, USA), 2 µL of cDNA (200 ng), and 2.5 µL of RNase-free water. The reactions were performed according to the following thermal profile: 2 min at 50 °C, 10 min at 95 °C, and 40 cycles of 15 s at 95 °C and 1 min at 60 °C. *GADPH* (Assay ID: Hs03929097_g1) was used as the reference gene. The standard curve method was used to analyze the expression of *PD-L1*, *PD-L2*, and *GADPH* genes.

### 2.7. Statistical Analysis

Data were analyzed with Statistica 13 PL (StatSoft, Kraków, Poland) and GraphPad Prism 8 (GraphPad Software, San Diego, CA, USA). The Shapiro–Wilk test was used to assess data distribution. The *p*-value was below 0.05, so the null hypothesis was rejected, indicating the data significantly deviated from a normal distribution. Therefore, statistical analysis was performed using nonparametric tests. The difference between the groups, i.e., the group of EC patients, the control, and the reference group, was analyzed by the Mann–Whitney U test. Spearman’s rank correlation coefficient (R) was applied to measure the strength of the association between two ranked variables. The data are presented as the median and range (min.-max). A *p*-value of <0.05 was considered statistically significant.

## 3. Results

### 3.1. Percentage of Myeloid (BDCA-1^+^CD19^−^) and Plasmacytoid (BDCA-2^+^CD123^+^) Dendritic Cells Within PBMCs of EC Patients

**Figure 2:**
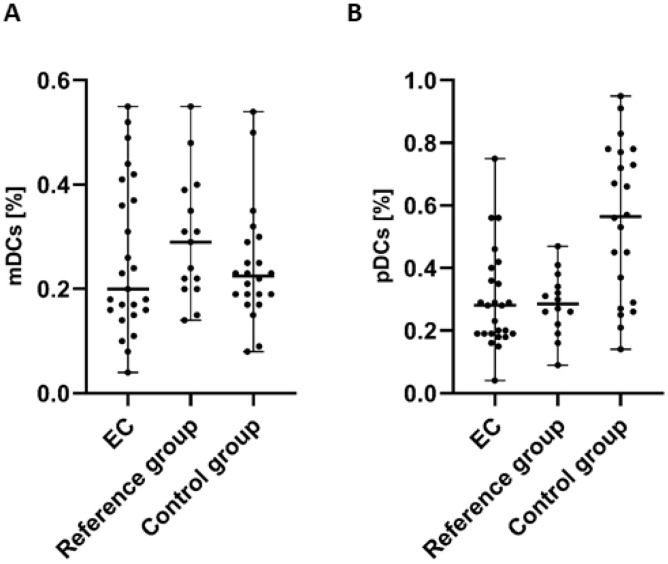
Percentage of mDCs (BDCA-1^+^CD19^−^ cells) (**A**) and pDCs (BDCA-2^+^CD123^+^ cells) (**B**) in PBMCs of EC patients (*n* = 25), the reference group (*n* = 15), and the control group (*n* = 22). Differences between groups were analyzed using the Mann–Whitney U test. Data are presented as median (range).

In the case of the examination of myeloid (BDCA-1^+^CD19^−^) dendritic cells (mDCs), we detected a lower percentage of BDCA-1^+^CD19^+^DCs in the PB of patients with endometrial cancer in comparison to the control group and the reference group. However, the differences did not reach statistical significance (Figure 2A).

The highest percentage of plasmacytoid (BDCA-2^+^CD123^+^) dendritic cells (pDCs) was detected in PB of the control group in comparison to PB of EC patients and the reference group. The percentage of BDCA-2^+^CD123^+^ cells in PB from EC patients was lower than in both the control and reference groups. However, similar to mDCs, the analysis of pDCs did not reveal statistically significant differences between groups (Figure 2B).

### 3.2. Percentage of Myeloid BDCA-1^+^CD19^−^ DCs with PD-L1 or PD-L2 Expression in Endometrial Cancer Patients

**Figure 3:**
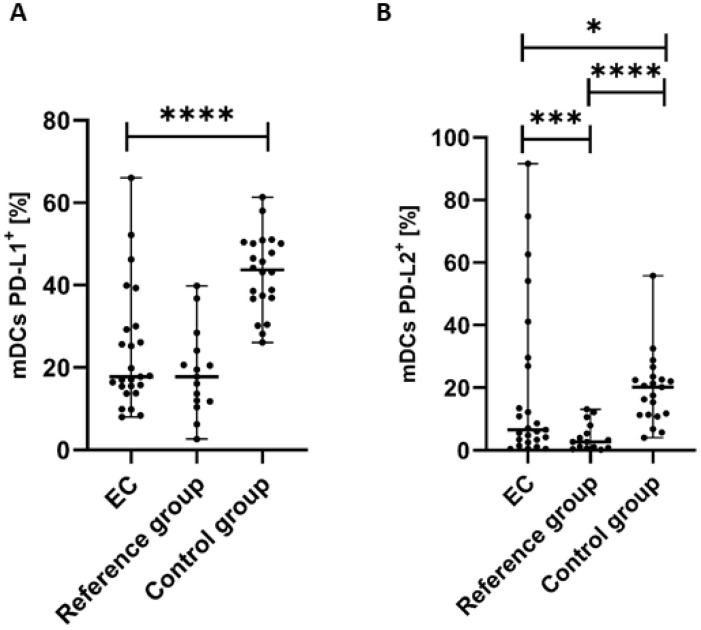
Percentage of BDCA-1^+^CD19^−^ cells with PD-L1^+^ (**A**) and PD-L2^+^ (**B**) in EC patients (*n* = 25), the reference group (*n* = 15), and the control group (*n* = 22). Differences between groups were analyzed using the Mann–Whitney U test. Data are presented as median (range), and statistically significant differences are indicated as: * *p* < 0.05, *** *p* < 0.001, **** *p* < 0.0001.

We observed statistically significant differences in the distribution of PD-L1 and PD-L2 expression on the surface of mDCs in the studied groups. The percentage of mDCs with PD-L1 expression in PB was significantly lower (*p* < 0.0001) in the endometrial cancer group than in the control group (Figure 3A). We detected a lower percentage of BDCA-1^+^CD19^−^PD-L1^+^ cells in the PB of EC patients compared to the reference group; however, the difference did not reach a significant level (Figure 3A). The percentages of PD-L2 positive mDCs was significantly lower in PB of EC patients than in the control group (*p* < 0.05) and in the reference group (*p* < 0.0001) (Figure 3B).

### 3.3. Percentage of Plasmacytoid BDCA-2^+^CD123^+^ DCs with PD-L1 or PD-L2 Expression in Endometrial Cancer Patients

**Figure 4:**
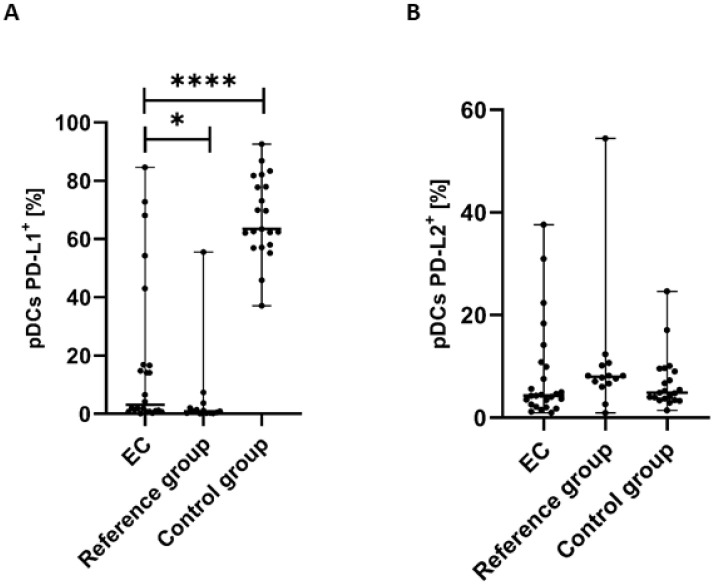
Percentage of BDCA-2^+^CD123^+^ cells with PD-L1^+^ (**A**) and PD-L2^+^ (**B**) in EC patients (*n* = 24), the reference group (*n* = 14), and the control group (*n* = 21). Differences between groups were analyzed using the Mann–Whitney U test. Data are presented as median (range), and statistically significant differences are indicated as: * *p* < 0.05, **** *p* < 0.0001.

The percentage of pDCs with PD-L1 expression was significantly lower in PB of patients with EC than in the control group (*p* < 0.0001) and the reference group (*p* < 0.05) (Figure 4A).

The percentage of BDCA-2^+^CD123^+^PD-L2^+^ cells in PB was higher in the reference group in comparison to EC patients and the control group. Moreover, we observed a higher percentage of the BDCA-2^+^CD123^+^PD-L2^+^ cells in the PB of the control group than in EC patients. However, the differences did not reach statistical significance (Figure 4B).

### 3.4. Relationship Between Clinical Data of EC Patients and Percentage of PB Myeloid DCs with PD-L1/PD-L2 Expression

**Figure 5:**
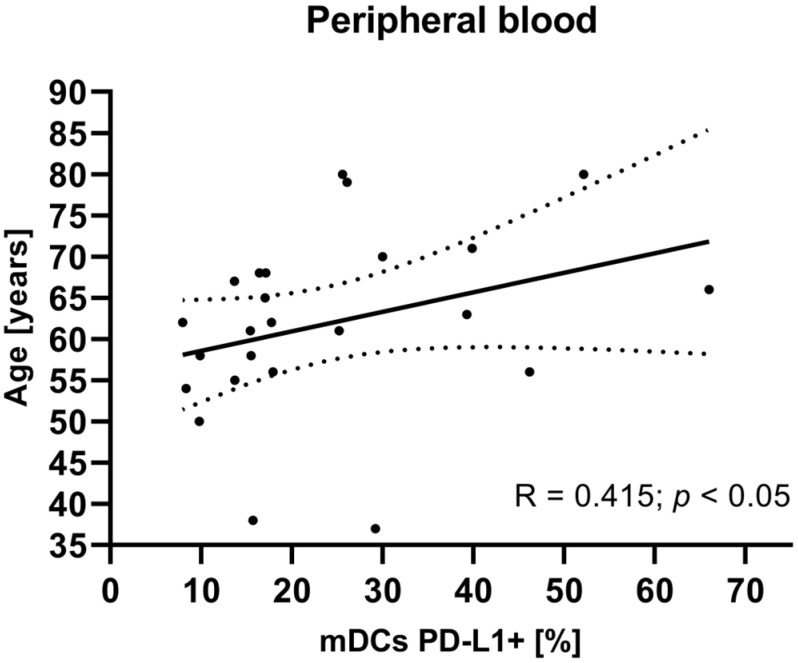
Relationship between the age of EC patients (*n* = 23) and the percentage of BDCA-1^+^CD19^−^PD-L1^+^ cells in peripheral blood. The association between variables was evaluated using Spearman’s correlation.

To investigate potential associations between the studied variables, we next examined the relationship between clinical parameters such as age, tumor grade, FIGO stage, and BMI of EC patients and mDCs and pDCs with PD-L1/PD-L2 expression. There were no statistically significant correlations between tumor grade, FIGO stage, and BMI in EC patients and the percentage of peripheral blood mDCs and pDCs with PD-L1/PD-L2 expression. The analysis revealed a positive correlation between the age of EC patients and the percentage of BDCA-1^+^CD19^−^PD-L1^+^ cells in plasma (R Spearman: 0.415; *t*(N-2) 2.091; *p* < 0.05) (Figure 5).

### 3.5. Percentage of MO (CD45^+^CD14^+^ Cells) and MO with PD-L1 and PD-L2 Epression in Endometrial Cancer Patients

**Figure 6:**
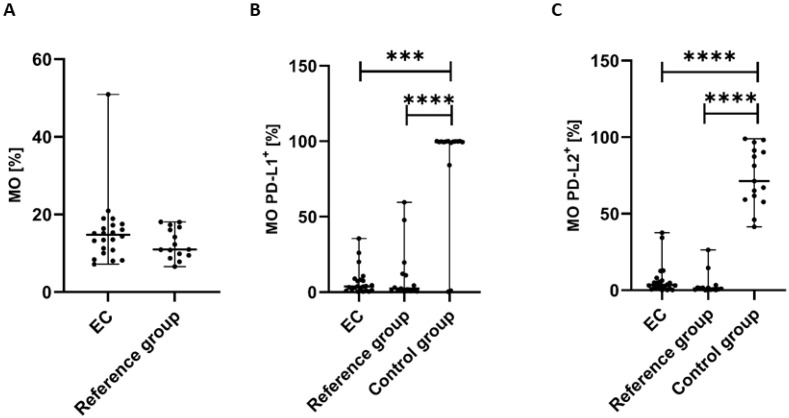
Percentage of CD45^+^CD14^+^ cells (**A**) in EC patients (*n* = 22) and the reference group (*n* = 15). Percentage of CD45^+^CD14^+^ cells with PD-L1 (**B**) and PD-L2 expression (**C**) in peripheral blood of EC patients, the reference group, and the control group (*n* = 15). Differences between groups were analyzed using the Mann–Whitney U test. Data are presented as median (range), and statistically significant differences are indicated as: *** *p* < 0.001, **** *p* < 0.0001.

The percentage of MO (CD45^+^CD14^+^ cells) was highest in the peripheral blood of EC patients in comparison to the reference group; however, the differences did not reach a significant level (*p* > 0.05) (Figure 6A).

The percentage of CD45^+^CD14^+^PD-L1^+^ cells in PB was significantly lower in EC patients than in the control group (*p* < 0.001) and the reference group (*p* < 0.0001) (Figure 6B). We also observed a higher percentage of MO with PD-L1 expression in PB of EC patients in comparison to the reference group; however, the differences did not reach statistical significance (Figure 6B).

The percentage of CD45^+^CD14^+^PD-L2^+^ cells was also significantly lower in PB of EC patients than in the control group and the reference group (*p* < 0.0001 and *p* < 0.0001, respectively) (Figure 6C). We also detected a higher percentage of MO with PD-L2 in PB from EC patients compared with the reference group; however, the difference did not reach statistical significance (Figure 6C).

### 3.6. Concentration of the Soluble Form of PD-1 (sPD-1), PD-L1 (sPD-L1), PD-L2 (sPD-L2) in Plasma of Endometrial Cancer Patients, the Control Group, and the Reference Group

**Figure 7:**
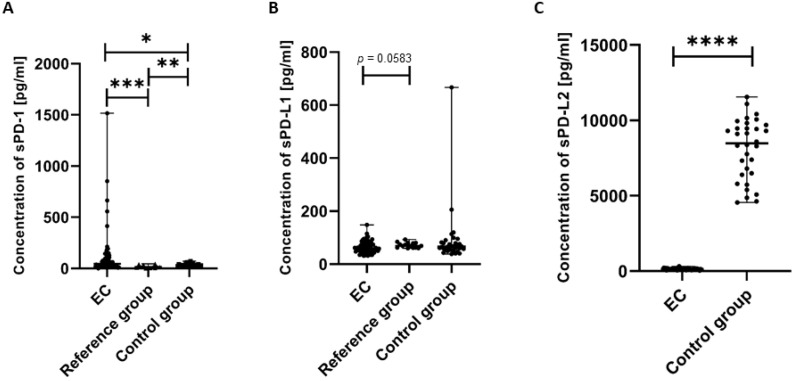
Levels (pg/mL) of sPD-1 (**A**), sPD-L1 (**B**), and sPD-L2 (**C**) in the plasma of EC patients (*n* = 73), the reference group (*n* = 12), and the control group (*n* = 32). Differences between groups were analyzed using the Mann–Whitney U test. Data are presented as median (range), and statistically significant differences are indicated as: * *p* < 0.05, ** *p* < 0.01, *** *p* < 0.001, **** *p* < 0.0001.

We next analyzed the concentration of the soluble forms of PD-1, sPD-L1, and sPD-L2 in plasma samples obtained from the study participants. We detected that the concentration of sPD-1 in the plasma of EC patients was significantly higher compared to the control group (*p* < 0.05) and the reference group (*p* < 0.001). We also observed that the level of sPD-1 in the plasma of the reference group was significantly higher than in the control group (*p* < 0.01) (Figure 7A).

The concentration of sPD-L1 in the plasma of EC patients was lower compared to the reference group; however, the observed difference narrowly missed statistical significance (*p* = 0.0583) (Figure 7B).

We also detected that the level of sPD-L2 in the plasma of EC patients was significantly lower than in the control group (*p* < 0.0001).

### 3.7. Relationship Between the Concentration of sPD-1, sPD-L1, sPD-L2 in Plasma and Different FIGO Stages and Histological Differentiation (Grading) of Endometrial Cancer

We next investigated whether the plasma concentration of sPD-1, sPD-L1, and sPD-L2 varies with histological differentiation and the clinical parameters, including age, tumor grade, FIGO stage, and BMI of EC patients. There were no statistically significant correlations between tumor grade or BMI in EC patients and the plasma concentrations of sPD-1, sPD-L1, and sPD-L2. The analysis revealed positive relationships between the FIGO stage of EC patients and sPD-L1 levels, and age of EC patients and sPD-1 levels.

#### 3.7.1. Assessment of the Relationship Between sPD-L1 Level in Plasma and Different FIGO Stages of EC Patients

**Figure 8:**
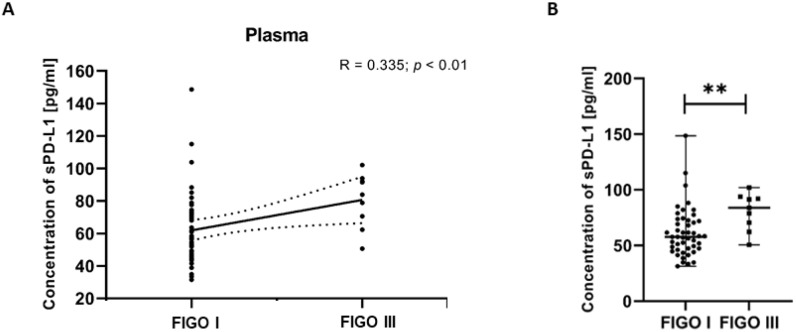
Relationship between the level of sPD-L1 in plasma and FIGO stages in EC patients (*n* = 55). The association between variables was evaluated using Spearman’s correlation (**A**). Levels (pg/mL) of sPD-L1 in the plasma of EC patients (*n* = 56) with different FIGO stages. Differences between groups were analyzed using the Mann–Whitney U test. Data are presented as median (range), and statistically significant differences are indicated as: ** *p* < 0.01 (**B**).

To investigate associations between clinical variables and immune parameters, we examined the relationships between FIGO stages of EC patients and plasma levels of sPD-L1. The analysis revealed a positive correlation between the sPD-L1 level in the plasma and the FIGO stages in EC patients (R Spearman: 0.335; *t*(N-2) 2.588; *p* < 0.01) (Figure 8A). We detected that the plasma sPD-L1 concentration in EC patients with FIGO stage III was significantly higher than in those with FIGO stage I (*p* < 0.01) (Figure 8B).

#### 3.7.2. Assessment of the Relationship Between sPD-1 Level in Plasma and Different FIGO Stages of EC Patients

**Figure 9:**
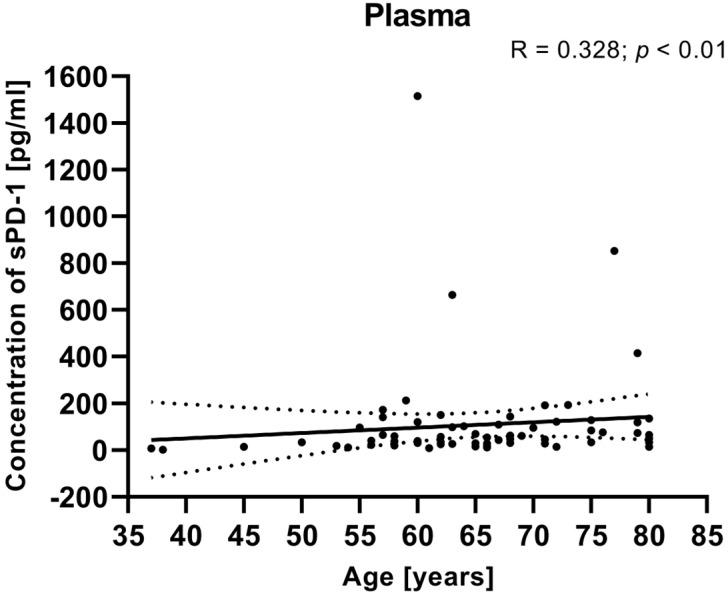
Relationship between the level of sPD-1 in plasma and age of EC patients (*n* = 68). The association between variables was evaluated using Spearman’s correlation.

We also calculated the relationship between the clinical data of EC patients (such as age, BMI, FIGO stage, and tumor grade) and the plasma levels of sPD-1. There was no significant correlation between the plasma concentrations of sPD-1 and clinical parameters (including FIGO stage, BMI, tumor grade). However, the analysis showed that a positive relationship between the sPD-1 level in the plasma and the age of EC patients (R Spearman: 0.328; *t*(N-2) 2.823; *p* < 0.01) (Figure 9).

### 3.8. Expression Levels of PD-L1 and PD-L2 Genes in Endometrial Cancer and the Reference Group Tissues

**Figure 10:**
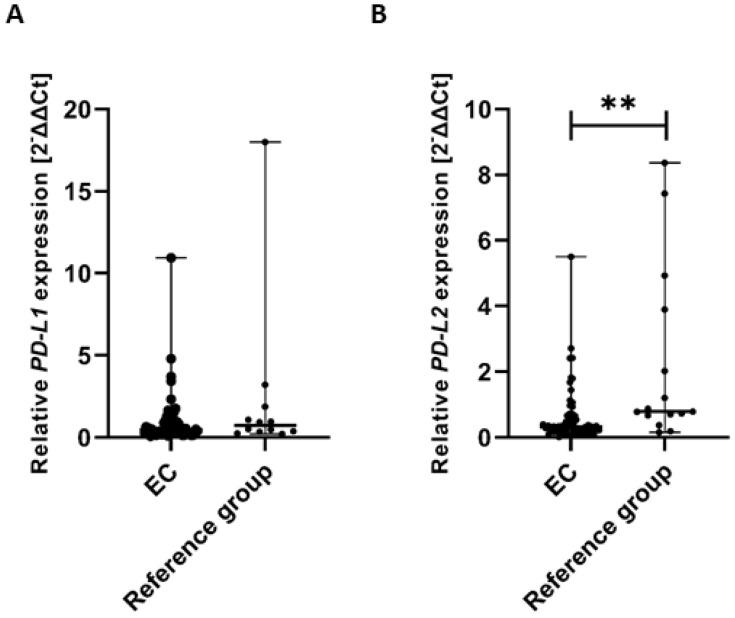
Comparison of *PD-L1* (**A**) and *PD-L2* (**B**) gene expression levels in tissue samples between EC patients (*n* = 61) and the reference group (*n* = 15). Differences between groups were analyzed using the Mann–Whitney U test. Data are presented as median (range), and statistically significant differences are indicated as: ** *p* < 0.01.

To assess whether there were significant differences in the expression of *PD-L1* and *PD-L2* genes in tissue samples from EC patients and the reference group, we performed the Mann–Whitney U test to compare both groups. The analysis revealed no statistically significant difference in *PD-L1* gene expression between EC patients and the reference group. Specifically, the median expression level of the *PD-L1* gene (calculated as 2^−ΔΔCt^) was significantly lower in EC patients compared to the reference group (U = 282.00) (Figure 10A).

The analysis revealed a statistically significant difference in *PD-L2* gene expression between EC patients and the reference group. Specifically, the median expression level of the *PD-L2* gene (calculated as 2^−ΔΔCt^) was significantly higher in the reference group compared to EC patients (U = 258.00, *p* < 0.01). These findings suggest a differential regulation of *PD-L2* gene expression between the two groups, which may have biological and clinical relevance (Figure 10B).

Gene expression levels were normalized to the reference gene as described in the Methods section.

### 3.9. Assessment of the Relationship Between Clinical Data of EC Patients and PD-L1/PD-L2 Gene Expression Level in Tissue Samples

**Figure 11:**
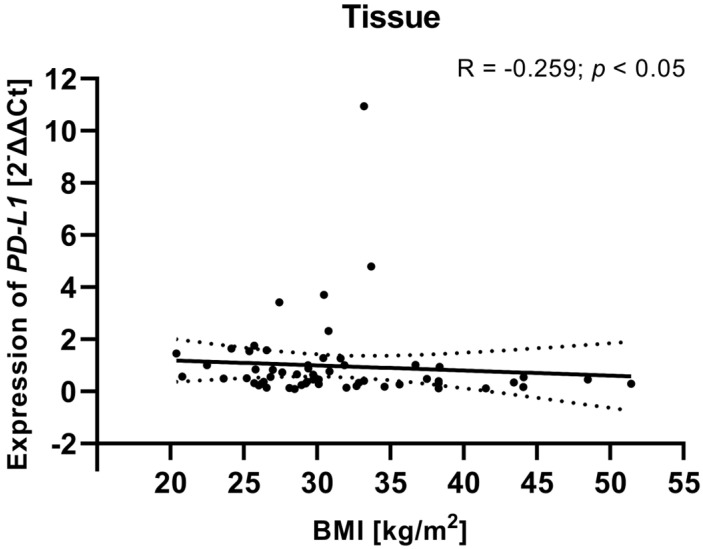
Relationship between the BMI of EC patients (*n* = 58) and *PD-L1* gene expression level in tissue samples. The association between variables was evaluated using Spearman’s correlation.

To investigate potential associations between clinical variables and gene expression, we examined the relationship between the clinical parameters (including FIGO stage, tumor grade, age, and BMI) of EC patients and *PD-L1/PD-L2* gene expression levels in tissue samples from EC patients. Analysis showed that a weak negative relationship between the BMI of EC patients and *PD-L1* gene expression level in tissue samples (R Spearman: −0.259; *t*(N-2) −2.01; *p* < 0.05) (Figure 11). Although the correlation was weak, the negative association may suggest that higher BMI is linked to lower *PD-L1* gene expression in EC tissues. The analysis revealed no statistically significant correlations for *PD-L2* or for the other clinical parameters. The lack of relationships between *PD-L2* expression and clinical parameters may indicate that *PD-L2* functions independently of these clinical features or is regulated by other mechanisms within the TME.

## 4. Discussion

Endometrial cancer is one of the most frequently diagnosed gynecological malignancies in developed countries [34,35]. The number of new EC cases continues to rise, and forecasts predict that by 2045, global incidence will increase by nearly 70% [1,5]. These alarming statistics highlight the complex biology and heterogeneity of EC, as well as the role of immune system dysfunction. Negative co-stimulation via the PD-1/PD-L1/PD-L2 axis is one mechanism that facilitates cancer cell survival. Within the tumor microenvironment (TEM), immune checkpoints (ICPs) deliver inhibitory signals to T lymphocytes, promoting immunosuppression.

In our study, we analyzed the expression level of PD-1 ligands—PD-L1 and PD-L2 - on antigen-presenting cells (APCs), including myeloid and plasmacytoid DCs, and monocytes in peripheral blood, in three study groups: EC patients, the control group, and the reference group. We also examined the concentration of soluble forms for sPD-1, sPD-L1, and sPD-L2 in plasma. Additionally, we analyzed the expression levels of *PD-L1* and *PD-L2* genes in tumor tissue from EC patients and the reference group. The obtained results were consolidated with clinical data of EC patients and then compared with data obtained from the group of healthy donors and the reference group. To the best of our knowledge, this is the first study to evaluate PD-L1 and PD-L2 expression on myeloid and plasmacytoid dendritic cells, as well as on monocytes, in patients with endometrial cancer using flow cytometry. It is noteworthy that previous studies of PD-L1/PD-L2 expression in EC have relied primarily on techniques such as immunohistochemistry (IHC) and immunofluorescence (IF), which analyzed tissue samples and characterized infiltrating cells as immune cells without explicit phenotyping.

We demonstrated differences in the distribution of mDCs (BDCA-1^+^CD19^−^ cells), pDCs (BDCA-2^+^CD123^+^ cells), and MO (CD45^+^CD14^+^ cells) with PD-L1 and PD-L2 expression among the studied groups. In patients with EC, we observed a significantly lower percentage of BDCA-1^+^CD19^−^ cells with PD-L1 expression compared to the control group. Similarly, the percentage of BDCA-1^+^CD19^−^PD-L2^+^ cells was significantly lower in patients with EC compared to both the control and reference groups. Our study also showed significantly lower percentage of CD45^+^CD14^+^ monocytes with PD-L1 and PD-L2 expression in EC patients compared to the control group.

The PD-1/PD-L1/PD-L2 axis plays a key role in inhibiting the immune response of cancer cells, enabling them to evade host immune surveillance. This mechanism promotes endometrial cancer cells’ proliferation and their adaptation to resistance to immune mechanisms [15,19]. However, there are now voices suggesting that this pathway does not always have to be associated with immunosuppression. Under certain conditions, PD-1^+^ T cells retain or even enhance their cytotoxic functions (including granzyme B secretion and IFN-γ production) as well as their proliferative capacity [36]. This highlights the fact that the effects of PD-1/PD-L1/PD-L2 may be context-dependent, depending on coinhibitory receptors or TME factors. Targeting PD-1/PD-L1/PD-L2 in combination with strategies aimed at TME-dependent suppression may optimize the outcomes of immunotherapy. Modern immunotherapeutic drugs such as Pembrolizumab and Dostarlimab, which belong to the checkpoint inhibitors (ICIs), revolutionize the treatment of EC by blocking the interaction of the PD-1 receptor with its ligands (PD-L1, PD-L2), leading to the restoration of T lymphocyte activity and enhanced anti-tumor response [27,31,37,38,39]. This represents a promising approach to the treatment of patients with EC [15,19,38].

Furthermore, in our study, we detected differences in the concentrations of the soluble form of the PD-1 receptor and its ligands in the plasma of the study groups. We observed an accumulation of sPD-1 in the plasma of patients with endometrial cancer compared to the control group. Simultaneously, we demonstrated significantly lower levels of sPD-L1 and sPD-L2 in EC patients. Contrary results were obtained by Mamat et al. [20], who observed accumulations of sPD-L1 and sPD-L2 in EC patients compared to the control group, and no significant differences between the study groups with respect to sPD-1 concentration. In turn, in the study conducted by Sulaiman et al. [40], it was observed that the mean plasma level of sPD-L1 in EC patients was significantly higher than in healthy women. In contrast, the mean plasma level of sPD-L2 was significantly lower in EC patients than in healthy women, consistent with our results. These discrepancies may result from differences in the structure of the study cohorts (e.g., stage of progression, histopathological type of tumor) as well as from the use of different methodological approaches [20]. It is worth emphasizing that in our study, we assessed the levels of sPD-1, sPD-L1 and sPD-L2 in numerous groups of patients with EC (*n* = 78) and in the control group (*n* = 32), while in Mamat et al. [20] the groups were much smaller (*n* = 23 and *n =* 11, respectively), and the study by Sulaiman et al. [40] included *n* = 11 EC patients and *n* = 19–20 healthy women. Interestingly, we observed a correlation between lower levels of sPD-L2 in plasma and reduced *PD-L2* expression in tissue among patients with endometrial cancer.

One of the aims of our study was to investigate the relationships between mDCs, pDCs, and MO expressing PD-L1 and PD-L2, the plasma levels of sPD-1, sPD-L2 and sPD-L2, as well as the gene expression levels of *PD-L1* and *PD-L2* in tissue, with the clinical features of patients with endometrial cancer. Interestingly, our results revealed significant correlations between the percentage of PD-L1-positive mDCs and the age of EC patients. Similarly, the plasma sPD-1 levels significantly correlated with the age of EC patients studied, which is consistent with previous findings reporting an association between sPD-1 levels and patients’ age [20]. However, this issue requires further investigation. A similar relationship was demonstrated by Wahba et al. [41]. In their study using IHC, they demonstrated a correlation between older patients’ age and positive PD-L1 expression in both tumor and tumor-infiltrating cells, although these results are not clearly confirmed in the literature. Considering advanced age (55–60 years), which is one of the most significant risk factors for the development of EC, the observed correlations may reflect age-related changes in the immune response. Additionally, they may indicate the effects of prolonged exposure to estrogens, which may influence the tumor microenvironment and promote its progression, thus confirming that age is a significant prognostic factor in EC [17,36,42].

In our study, we observed a positive correlation between plasma sPD-L1 levels in EC patients and the clinical stage of disease. Higher sPD-L1 levels were particularly significant in patients with an advanced stage of EC according to the FIGO classification (II-III). Similar observations were reported by Mamat et al. [20], who demonstrated that serum sPD-L1 levels were significantly higher in EC patients in comparison to the control group, and were further elevated in cases with advanced disease. Both our results and the literature data indicate a correlation between plasma and serum sPD-L1 levels and the clinical stage of EC. These observations suggest that sPD-L1 may play a role as a potential prognostic biomarker in EC, reflecting the degree of disease progression and aiding risk assessment and monitoring of the clinical course of the disease.

What is interesting in our study is that we observed a negative correlation between *PD-L1* gene expression levels and BMI of EC patients, where higher BMI was associated with lower *PD-L1* expression levels.

It is clear that obesity plays a significant role in the pathogenesis of EC and is considered a strong risk factor for EC, often coexisting with other factors, such as age and metabolic syndrome, which influence the development of the tumor and its TME [43,44,45,46]. High BMI, with both overweight (BMI 25–29.9) and obesity (BMI ≥ 30), significantly increases the risk of EC. High levels of adipose tissue provide an ideal environment for the overproduction of estrogen in adipocytes, promoting the process of endometrial carcinogenesis [43,44,47,48]. The majority of EC patients are diagnosed after menopause (statistical age of onset is around 60 years), which, together with increased BMI and prolonged exposure to estrogens, favors the development of immunosuppression in the tumor microenvironment [36,43,48]. Furthermore, obesity is associated with chronic inflammation and elevated levels of cytokines, such as TNF-α, interleukin-1 beta (IL-1β), interleukin-6 (IL-6), and adipokines (e.g., leptin), which support tumor cell proliferation, angiogenesis, and TME modulation [46]. Probably, obesity can modify the expression of ICPs, including PD-L1 [43,49,50].

These findings may indicate that obesity alters TME, shifting immune regulation from the local to the systemic level. In obese individuals, chronic low-grade inflammation and adipokine signaling may lead to T cell dysfunction and exhaustion, which is characterized by increased PD-1 expression on circulating CD8^+^ T cells rather than increased tumor *PD-L1* expression. Consequently, although tumors in obese patients may exhibit lower *PD-L1* expression and reduced CD8^+^ T cell infiltration, exhausted T cells may be more effectively reactivated by PD-1 blockade. This phenomenon is supported by the concept of the ‘obesity paradox’, in which obesity, despite promoting tumorigenesis and immune dysregulation, paradoxically increases the efficacy of ICIs [43]. Therefore, lower *PD-L1* expression in obese EC patients does not necessarily reflect an altered immune system structure that favors response to PD-1/PD-L1-based immunotherapy. Obesity, therefore, plays a dual role; on the one hand, it promotes the development and progression of cancer through metabolic and immunological disorders, and on the other hand, it may increase the effectiveness of immunotherapies based on PD-1/PD-L1 checkpoint inhibitors [51]. Adipose tissue, by participating in the conversion of androgens to estrogens, promotes increased endometrial proliferation and neoplastic transformation. Although the available literature does not provide definitive evidence for this relationships, Dyck et al. [49] suggests that higher BMI in EC patients may be associated with reduced *PD-L1* expression in TME. Furthermore, in EC patients, they also found an inverse correlation between BMI and CD8^+^ T cell infiltration in the tumor. However, this publication does not clarify *PD-L1* expression levels at the tumor level in the context of obesity.

Our study indicates that dysregulation of the PD-1/PD-L1/PD-L2 pathway in EC is associated with clinicopathological features and may serve as a valuable source of therapeutic targets, as well as diagnostic and prognostic biomarkers. To the best of our knowledge, this is the first study to comprehensively investigate the PD-1/PD-L1/PD-L2 axis in endometrial cancer by combining gene expression profiling in tumor tissue with the assessment of surface expression on immune cell subsets and the measurement of soluble forms in plasma. This multi-level approach provides novel insights into the role of this pathway in disease progression and its potential diagnostic and prognostic significance. The observed correlations between immunological parameters and clinical features (including age, BMI, and FIGO stage) further support the translational relevance of our findings. Our study has certain limitations, primarily related to the age-matching of the healthy control group to EC patients. Recruiting healthy postmenopausal women over the age of 60 as blood donors is challenging. To address this limitation, we included a reference group consisting of patients with Leiomyoma, which allowed us to obtain samples from women with benign tumors of the uterus of similar age to those with EC. However, it might have been more appropriate to include patients without any uterine or ovarian pathology, as suggested by Mamat et al. [20] in their study. Future studies should validate these findings in larger, independent cohorts and in preclinical models, incorporating longitudinal and functional approaches to explore the mechanistic and therapeutic implications of PD-1/PD-L1/PD-L2 pathway dysregulation in endometrial cancer.

## 5. Conclusions

In conclusion, the reduced percentages of PD-L1/PD-L2 positive APCs, together with altered concentrations of their soluble forms and correlations with gene expression in TT, suggest that dysregulation of the PD-1/PD-L1/PD-L2 pathway may impair the ability of the immune system to control endometrial cancer growth effectively. 

The associations observed between immunological parameters (e.g., sPD-L1, *PD-L1* expression) and clinical features (such as BMI, FIGO stages), highlight the diagnostic and prognostic potential of these factors and their possible relevance in future therapeutic strategies. However, to fully elucidate the biological implications of these findings and validate the proposed hypothesis, further comprehensive functional studies are warranted. In future research, we intend to expand and deepen our analyses by incorporating this aspect through retrospective molecular assessments (POLE, p53, MSI, dMMR), which may provide further insight into their clinical relevance.

## Figures and Tables

**Table 1:** Clinical characteristics of patients with EC.

The Clinical Features	Endometrial Cancer Patients (*n* = 78)
Age (median), years (range)	65 (37–82)
Age of menarche (means, range)	14 (9–18)
Age of menopause (means, range)	51.5 (37–58)
FIGO Stages, *n* (%)	
Early (IA-IB)	55 (85%)
Advanced (III)	10 (15%)
Grade of Cancer, *n* (%)	
G1	23 (30%)
G2	51 (66%)
G3	3 (4%)
Body Mass Index (BMI) (median, range)	30.3 (19.3–51.4)
BMI category, *n* (%)	
Normal	7 (9%)
Overweight	29 (36%)
Obese	44 (55%)
Healthy group (*n* = 32)	
Age (median), years (range)	32 (25–45)
Reference group (*n* = 15)	
Age (median), years (range)	48 (31–78)

## Data Availability

All data generated or analyzed during this study are included in this publication.

## Abbreviations

The following abbreviations are used in this manuscript:

EC	Endometrial cancer
BMI	Body Mass Index
WHO	World Health Organization
ASR	Age-standardized rate
PCOS	Polycystic Ovary Syndrome
TME	Tumor microenvironment
DCs	Dendritic cells
IL-10	Interleukin-10
IL-6	Interleukin-6
IL-1β	Interleukin-1 beta
Treg	Regulatory T cell
mDCs	Myeloid dendritic cells
TNF-α	Tumor necrosis factor alpha
pDCs	Plasmacytoid dendritic cells
ICPs	Immune checkpoints
ICIs	Immune checkpoint inhibitors
PD-1	Programmed cell death protein 1
PD-L1	Programmed death-ligand 1
PD-L2	Programmed death-ligand 2
FIGO	The International Federation of Gynecology and Obstetrics
PB	Peripheral blood
TT	Tumor tissue
mABs	Monoclonal antibodies
FMO	Fluorescence Minus One
sPD-1	Soluble form of PD-1
sPD-L1	Soluble form of PD-L1
sPD-L2	Soluble form of PD-L2
qPCR	Quantitative polymerase chain reaction
APC	Antigen-presenting cell
IHC	Immunohistochemistry
IF	Immunofluorescence
FDA	U.S. Food and Drug Administration
PBMC	Peripheral Blood Mononuclear Cell
